# Dysregulated metabolism and the regulation of aromatase in breast adipose stromal cells in obesity and cancer

**DOI:** 10.1186/1753-6561-6-S3-P9

**Published:** 2012-06-01

**Authors:** Nirukshi U Samarajeewa, Fangyuan Yang, Maria Docanto, Minako Sakurai, Hironobu Sasano, Evan R Simpson, Kristy A Brown

**Affiliations:** 1 Metabolism & Cancer Laboratory, Prince Henry’s Institute, Clayton, Victoria, 3168, Australia; 2 Department of Physiology, Monash University, Clayton, Victoria, 3168, Australia; 3 Depatment of Pathology, Tohoku University, Sendai, Japan; 4 Department of Biochemistry & Molecular Biology, Monash University, Clayton, Victoria, 3168, Australia

## Background

The risk of breast cancer in postmenopausal women is increased two-fold with obesity, and the majority of breast tumours that arise are oestrogen-dependent. After menopause, when the ovaries cease to produce oestrogens, it is the local production of oestrogens within the breast adipose stromal cells (ASCs) which promotes and sustains tumour growth. This is largely due to the increased expression of aromatase, responsible for the conversion of androgens to oestrogens. Aromatase expression in breast cancer is known to be under the control of a proximal promoter, promoter PII, which is maximally activated by cAMP-dependent mechanisms. We have previously demonstrated that the LKB1/AMPK pathway is a key negative regulator of aromatase expression within the breast by inhibiting the nuclear translocation of the CREB co-activator CRTC2 [1]. We have also demonstrated that the tumour-derived factor prostaglandin E2 (PGE2) and the obesity-associated factor leptin stimulate aromatase expression by inhibiting LKB1 and AMPK expression and activity. Hypoxia inducible factor-1α (HIF1α) is emerging as a potent regulator of glycolysis in tumour cells and we have identified a putative hypoxia response element in aromatase promoter Pll immediately adjacent the cAMP response element, known to be bound by the CREB-CRTC2 complex in ASCs in breast cancer. We therefore hypothesise that HIF1α may be involved in regulating aromatase in breast cancer.

## Materials and methods

Primary human breast ASCs were isolated from tissue after breast reduction surgery. Real-time PCR, Western blotting, immunofluorescence and high content screening were used to assess HIF1α expression and localisation after PGE2 treatment. Chromatin immunoprecipitation (ChIP) was performed to examine the interaction of HIF1α with aromatase promoter PII and reporter assays were performed to assess the effect of HIF1α on PII activity. Double immunohistochemistry for HIF1α and aromatase was also performed on sections of formalin-fixed paraffin-embedded breast tissue from breast cancer patients and cancer-free patients.

## Results

We have found that PGE2 increases HIF1α transcript expression, nuclear localisation and binding to aromatase PII in primary human breast ASCs. Moreover, HIF1α causes a significant increase in PII activity and acts cooperatively with CREB to cause this induction. Data from breast cancer patient samples demonstrates that HIF1α is also increased in tumour-associated ASCs compared to breast tissue from cancer-free women. Interestingly, the majority of ASCs from breast cancer patient samples display staining for both HIF1α and aromatase.

## Conclusions

This study is part of a growing body of evidence indicating that dysregulated metabolism is not only a characteristic of adipocytes in obesity and epithelial cells in cancer, but also occurs in tumour-associated ASCs. We demonstrate that dysregulation of metabolic pathways is accompanied by an increase in aromatase expression within the breast adipose and provide an additional mechanism whereby obesity is linked to breast cancer. Clinical studies are currently underway to explore the use of drugs which target these pathways, such as metformin, for their use as novel aromatase inhibitors for the prevention and treatment of postmenopausal breast cancer.
